# Through An Equity Lens: Illuminating The Relationships Among Social Inequities, Stigma And Discrimination, And Patient Experiences of Emergency Health Care

**DOI:** 10.1177/00207314221075515

**Published:** 2022-01-31

**Authors:** Colleen Varcoe, Annette J. Browne, Vicky Bungay, Nancy Perrin, Erin Wilson, C. Nadine Wathen, David Byres, Elder Roberta Price

**Affiliations:** 1 120487 University of British Columbia (UBC), Vancouver BC, Canada; 2 1466 Johns Hopkins University, Baltimore, Maryland, USA; 3 6727 University of Northern British Columbia (UNBC), Prince George, British Columbia, Canada; 4 104272 Western University, London. Ontario, Canada; 5 Provincial Health Services Authority, Vancouver, British Columbia, Canada

**Keywords:** intersectionality, stigma, discrimination, emergency services, equity, patient reported experiences, repeat use

## Abstract

People who experience the greatest social inequities often have poor experiences in emergency departments (EDs) so that they are deterred from seeking care, leave without care complete, receive inadequate care, and/or return repeatedly for unresolved problems. However, efforts to measure and monitor experiences of care rarely capture the experiences of people facing the greatest inequities, experiences of discrimination, or relationships among these variables. This analysis examined how patients’ experiences, including self-reported ratings of care, experiences of discrimination, and repeat visits vary with social and economic circumstances. Every consecutive person presenting to three diverse EDs was invited if/when they were able to consent; 2424 provided demographic and contact information; and 1692 (70%) completed the survey. Latent class analysis (LCA) using sociodemographic variables: age, gender, financial strain, employment, housing stability, English as first language, born in Canada, and Indigenous identity, indicated a six-class solution. Classes differed significantly on having regular access to primary care, reasons for the visit, and acuity. Classes also differed on self-reported discrimination every day and during their ED visit, ratings of ED care, and number of ED visits within the past six months. ED care can be improved through attention to how intersecting forms of structural disadvantage and inequities affect patient experiences.

Emergency departments (EDs) in Canada are often overcapacity and operate under considerable pressure^1^. Efforts to address ED strain have focused on diverting people from using EDs for problems that could be addressed in primary care settings^2^ and on increasing primary care capacity with interventions including case management care plans and diversion strategies^3,4^. Consequently, EDs and ED staff are positioned to apply standard triage criteria; to judge who is/is not deserving of and appropriate for ED care; and, in the process, to make efforts to deter those judged undeserving or inappropriate^5^. However, primary care capacity remains inadequate to meet population needs, creating significant tension for those working in and responsible for administering ED care. Indeed, in describing revisions to the Canadian Triage Assessment Scale (CTAS), Bullard and colleagues (^5^, p. S21) warn that “recently a number of administrators have sought to co-opt CTAS as a tool to identify “inappropriate ED visits,” with plans to divert them away from the ED. In addition, retrospective reviews of discharged ED patients have attempted to define “primary care appropriate” ED diagnoses and calculate the percent of patients who are “misusing” the EDs.

Primary care responsiveness and capacity are particularly impactful for people who experience significant health and social inequities, including people living in rural settings, Indigenous people, people who are homeless, and marginalized people who use drugs^6,7–10^. This literature confirms, repeatedly, that people who face the greatest structural^1^ disadvantages—and social and health inequities—have greatest challenges receiving primary care services that align with their needs and, consequently, may have to rely on EDs when seeking care, thus potentially presenting with lower acuity and judged as “inappropriate.” Further, people who experience the greatest inequities tend to experience intersecting forms of stigma, discrimination, and negative social judgment in the wider social world and in the health care sector, including in EDs. Although judgments regarding deservedness for ED care are intended to be made based on acuity and standard criteria, social judgments influence health care policies and practices. Small-scale studies of groups experiencing marginalization as a consequence of structural stigma and discrimination show that people who experience the greatest social and health inequities often have poor experiences in EDs so that they are deterred from seeking care, leave before care is complete, receive inadequate care, face a lack of follow-up care in the community, and/or return repeatedly as health issues are unresolved^11–16^. Thus, health and health care inequities are exacerbated by the gaps in care provided at EDs. However, larger scale efforts to measure and monitor patient experiences of care (PEOC) in EDs rarely disaggregate by social circumstances and seldom capture experiences that reflect structural inequities, including barriers to care, experiences of discrimination, or the relationships among these variables, thus providing limited direction to mitigate inequities^17^.

Within the context of a study to develop and test an intervention to promote health equity in EDs^18^, we sought to establish a baseline of patient perspectives of care. The purpose of the analysis presented in this article was to better understand the ED experiences of people from the widest possible range of social circumstances. We identified how patients’ experiences of care—including whether or not they had regular access to a source of primary care (a “primary care home”) such as a primary care physician, nurse practitioner, or clinic; why they attended the ED; their triaged acuity; their self-reported ratings of care; experiences of discrimination; and repeat visits—vary with their experiences of social inequities, stigma, and discrimination, experiences that reflect structural inequities. Our aim is to illuminate how intersecting forms of health and social disadvantages affect experiences of care and to use this analysis to generate recommendations regarding strategies and actions to enhance the capacity of EDs to mitigate ongoing inequities—particularly for people who experience oppressive structural and social circumstances.

The research questions that informed this analysis were:

1. Do people cluster into unique groups based on their pattern of structural advantages/disadvantages, as measured by financial strain, housing instability (current housing), age, gender, not being born in Canada, English as a first language, identifying as Indigenous, being employed, and having accessed a shelter in the past 12 months?
2. Do the groups differ on having regular access to primary care, the reason for attending ED, or acuity rating?
3. Do these groups differ on patient ratings of care, their self-reported experiences of discrimination in EDs, everyday experiences of discrimination, or self-reported number of ED visits within the past six months?

Based on these analyses, we consider the implications for EDs with regard to their role and responsibility in responding to health and social inequities, particularly in relation to people who are most significantly affected.

## EQUIP Emergency

Building on two decades of research on violence and inequity in health care, with particular attention to Indigenous people’s^2^ experiences of health and health care, we designed and tested an organizational intervention (Equipping Health Care for Equity – EQUIP) in primary health care settings^19^. The results were promising^20–22^, and because the findings suggested that emergency settings were a key site for promoting equity, we determined to adapt EQUIP to EDs. Researchers, Indigenous and health care leaders, and staff in three EDs partnered to adapt, enhance, and test the intervention (EQUIP ED) and refine a framework to promote equity in EDs. The study, fully described elsewhere^18^, was conducted within the Canadian province of British Columbia, in the EDs of the University Hospital of Northern British Columbia, a small regional hospital serving rural and remote communities over a large area; a larger, urban hospital in Vancouver (St. Paul's Hospital); and the largest ED in the province in Surrey Memorial Hospital, which serves diverse suburban communities. To identify whether the intervention resulted in changes over time, we needed to understand the experiences of care of patients accessing the ED and sought to establish a baseline pre-intervention. This provided an opportunity to examine how patients’ experiences of care vary with experiences that reflect structural inequities.

## Theoretical Lenses

The EQUIP ED study and this analysis are guided by critical theoretical approaches to health equity and intersectionality. The World Health Organization defines equity as “the absence of avoidable, unfair, or remediable differences among groups of people, whether those groups are defined socially, economically, demographically, or geographically or by other means of stratification. “Health equity” or “equity in health” implies that ideally everyone should have a fair opportunity to attain their full health potential and that no one should be disadvantaged from achieving this potential^23^.”

Examining equity in health care requires attention to those at greatest risk of poor health, including those most affected by the negative impacts of structural inequities such as poverty, lack of affordable housing, stigma, racism, and other forms of discrimination. Using an equity lens guided us to: (*a*) develop our data collection approaches toward optimal inclusion and (*b*) seek to analyze ED experiences of care for differences among groups.

The way societies are arranged through policy and social stratification shapes access to social and material resources, including income, housing, and employment. In Canada, a former British colony and liberal welfare state with an above-average poverty rate compared to other Organisation for Economic Co-operation and Development countries, key policies shaping the experiences of population groups include economic, housing, and immigration policies and those stemming from the Indian Act (1885)^24^ to govern Indigenous peoples. However, people experience inequities at the confluence of multiple, intersecting structural arrangements—that is, individuals occupy multiple social locations and belong to multiple groups simultaneously. For example, although Indigenous people in Canada face disproportionate structural disadvantage, not all live in poverty, not all are subject to federal policies that limit property ownership, and not all have limited access to education. An intersectional lens guided us to seek an approach to analyze data beyond single variables of ethnocultural identity or social location to examine intersecting social categories.

Emerging from black feminist scholarship^25–27^, intersectionality offers a theoretical and analytical approach to understanding how multiple forms of structural inequity interrelate. It helps to consider how interlocking systems of oppression such as racism, classism, and sexism disadvantage people based on their multifaceted social locations. Intersectionality challenges the primacy of any single category or additive categories of analysis, pointing toward understanding complexities of differences between and among individuals and groups, as well as increasing attention to structural inequities and operations of power across multiple domains^28,29^. Social locations, material circumstances, and ideological identities are understood as woven together by strands of intersecting systems of power and oppression at a range of individual, relational, and structural levels^30^. This contrasts with prevailing trends in research focused on examining PEOC through narrowly defined lenses. Whereas European countries routinely include socioeconomic data as part of health statistics to illustrate interactions among variables, in Canada and the United States, most public health and population-level surveillance studies remain focused on race-based categories and/or ethnicity as the primary variable of concern^31–33^. The net effect has been to obscure understandings of structurally mediated, pervasive patterning of health inequities—together with patients’ experiences of those inequities in health care contexts and as influenced by social inequities, issues of stigma, and discrimination^32^. Together, these theoretical lenses have implications for how data are collected, what data are collected, how data are analyzed, what we do with the data, and the processes by which these decisions are made.

## Method

Within a mixed-methods multisite design that included longitudinal collection of survey data from patients and staff at up to four time points prior to and after intervention work, observational field work, and interviews with staff^18^, patients presenting to each of the EDs were surveyed. The analysis presented in this article is based on survey data collected from patients prior to intervention work. Each adult patient was approached if they appeared able to be approached (eg, not undergoing treatment for life-threatening problems) and able to provide informed consent. Thus, there were no exclusion criteria other than being unable to provide consent. If patients were unable to give consent when first presenting for care (eg, unconscious, in pain) but became able subsequently, they were approached to participate when it appeared they were able to consent. We estimate that approximately 80% of presenting patients were invited to participate. Contact information and demographic data were collected from interested and consenting patients (Interview Part 1), who were then followed up immediately after discharge (but before they left the department), in their hospital room if admitted, or interviewed by phone within five days of their visit (Interview Part 2). Data were collected directly on tablets, with patients inputting the data if they preferred. Data collectors were explicitly trained in equity-oriented approaches to ensure effective, affirming communication, using strategies to help all participants feel respected, accepted, and not judged. Data were collected across all times of day and days of the week. Depending on hospital size, our research team required 12 to 25 days to achieve the required sample size. The interviews took an average of 10 to 15 min for each of Parts 1 and 2, with variability dependent on the patient's health state, need for linguistic interpretation, and interruptions for care.

### Measures

The measures used have been published elsewhere^18^. Because an equity lens focuses attention on structural conditions and influences on health, we used measures to serve as proxies for social inequities, including a measure of housing stability taken from the Canadian Community Health Survey^34^, a measure of financial strain^35^, maximally inclusive approaches to demographic variables such as gender^36^, and ethnicity items used in our previous research^21^. Financial strain was dichotomized into “somewhat” or “very difficult” versus “not very” or “not at all difficult” to live on current income. Living condition was classified as “stable” versus “precarious” based on their current situation. Because an equity lens also directs attention to forms of stigma and discrimination that disadvantage some and privilege others, we sought to survey patients about these experiences (see Table 1). Drawing on an intersectional lens and cognizant of the concern that quantitative studies of discrimination and health often focus on single axes of discrimination^37^, we used the Everyday Discrimination Scale^38^, which asks people to identify their perceptions of the diverse reasons they are being discriminated against. Emergency room-specific discrimination was measured as the number of items on which a patient felt discriminated against during the ED visit, using an adapted version of the Discrimination in Medical Settings Scale^39^. Patients’ ratings of their care during the visit were measured using 25 items from three sources: the Emergency Department Patient Experiences of Care (EDPEC) Scale^40^, the British Columbia EDPEC^41^, and seven items developed for the EQUIP Emergency study. The patient's CTAS rating^5^, which is the standard approach to classifying acuity in EDs (https://ctas-phctas.ca/), was obtained from triage staff or patient flow monitors, depending on the department. The CTAS ranges from 1 (the highest acuity, requiring immediate life-saving intervention) to 5 (the lowest acuity, usually classified as non-urgent). We also gathered information on whether each person had a usual primary care setting to receive care and the reason for the current ED visit.

**Table 1.** Measures of Discrimination and Experiences of Care.

Measures	Source and Reference	Items	Range
Discrimination in Everyday Life^38^	Everyday Discrimination Scale	9	0 to 5 Overall score: 0 to 45
Discrimination During ED Visit^39^	Discrimination in Medical Settings Scale	7	1 to 5 Overall score: 7 to 35
Experiences of Care	Emergency Department Patient Experiences of Care (EDPEC) Scale^40^ (includes the Quality of Care measure)	15	Quality of care: 0 to 10
	British Columbia EDPEC^41^	9	NA
	Investigator developed (EQUIP ED)	12	NA
Patient Acuity on Presentation	Canadian Triage Assessment Scale (CTAS) (5)	1	1 to 5

### Analysis

Drawing on an intersectional lens and in alignment with calls for quantitative analyses to “catch up” to intersectional theorizing^42^, we sought an inter-categorical approach to analysis. We addressed Research Question 1 to identify statistical classes of people with similar patterns of structural advantage/disadvantage using Latent class analysis (LCA). Nine dichotomous variables were included in the analysis (financial strain, stable living condition, age > 65, gender [11 people did not identify with either male or female], born in Canada, English as a first language, self-identification as Indigenous, employed, sheltered in the last year). Goodness of fit statistics (Akaike's information criterion [AIC] and Schwarz's Bayesian information criterion [BIC]) and interpretability were used to determine the number of classes. Next, we explored how the classes differed on access to care, reasons for seeking care, experiences of discrimination, number of ED visits, ED discrimination, and quality of care using general linear models. All models used a Gaussian distribution, except number of ED visits in the past six months, which used a Poisson distribution.

## Findings

### Sample

Consent, demographic data, and contact information (Part 1) were obtained for 2424 people, and complete data (Parts 1 & 2) were obtained for 1692 (70%) people, who were included in the analysis. Those who completed the survey were significantly older (*p* < 0.001) and more likely to be born in Canada (*p* = 0.004), to have English as a first language (*p* = 0.000), to be employed (*p* = 0.000), and to live in a stable situation (*p* = 0.001), and they were less likely to be Indigenous (*p* = 0.05) and to have accessed a shelter (*p* = 0.001) than those who did not complete the survey.

The efforts described above resulted in recruiting a sample that was highly diverse in terms of social location, with greater representation from people over 65, people accessing homelessness shelters, and Indigenous people than in the underlying provincial population (see Table 2). The sample was generally representative of the populations served by each ED. Compared to the sample obtained from emergency patients in the same province during a similar timeframe using mail-out surveys (column in Table 2, with comparisons with the British Columbia EDPEC), our sample tended to be younger and more highly educated, with poorer self-reported health and higher acuity. Similar to the findings by Chiu and colleagues^17^, the sample was more diverse in terms of having greater representation from Indigenous people and those less likely to have a primary care home.

**Table 2.** Demographic Characteristics of Patients Completing (N = 1692).

Variable	n (%) of EQUIP ED Sample	n (%) of BC EDPEC Sample	n (%) of BC Census Sample
**Canadian Triage & Acuity Scale (CTAS)**			NA
1 – Resuscitation	8 (0.6)	39 (0.3)	
2 – Emergent	323 (23.2)	2018 (16)	
3 – Urgent	680 (48.9)	5789 (45.9)	
4 – Less urgent	350 (25.2)	4023 (31.9)	
5 – Non-urgent	30 (2.2)	580 (4.6)	
**Age**	Range: 18 to 98, Mean: 51.47, SD: 18.621		Range: 0 to 100 + , Mean: 42.3, Median: 43.0^64^
**Age 65 and over**			
Under 65	1235 (73.5)	9530 (67.6)	3,799,070 (81.7)
Over 65	445 (26.5)	4546 (32.4)	848,985 (18.3)
**Gender**			
Woman	835 (49.3)	7568 (53.9)	2,369,815 (51.0)
Man	837 (49.7)	6506 (46.1)	2,278,245 (49.0)
Non-binary	11 (0.7)	1 (0)	N/A
**Education**			
Didn’t complete secondary school/high school	353 (21.1)	4341 (29.8)	601,640 (15.5)
Completed secondary school/high school	372 (22.2)	2835 (19.8)	1,138,565 (29.4)
Some or completed post-secondary	949 (56.7)	5972 (46.2)	2,130,175 (55.0)
**Born in Canada**			
No	412 (24.6)		1,292,675 (30.5)
Yes	1265 (75.4)		3,167,155 (69.5)
**First language English**			
No	368 (25.7)		1,428,305 (31.1)
Yes	1063 (74.3)		3,170,110 (68.9)
**Speaks English**			
Does not currently speak English	50 (3.0)		151,760 (3.4)
Currently speaks English	1641 (97.0)		4,442,695 (96.6)
**Indigenous**			
Non-Indigenous	1395 (83.5)	12116 (94.1)	4,289,655 (94.1)
Indigenous	275 (16.5)	1246 (5.9)	270,585 (5.9)
**Living situation – dichotomized**			N/A
Precarious housing^5^	175 (10.4)		
Stable housing	1505 (89.6)		
**Accessed a shelter in the past year**			N/A
No	1568 (93.8)		
Yes	104 (6.2)		
**Primary work status**			
Employed FT or PT	718 (43.0)		2,305,690 (59.6)
Unemployed	387 (23.2)		165,975 (4.3)
Retired	465 (27.8)		1,398,710 (36.1)
Other (includes seasonal, exchange services or student)	100 (6.0)		
**Receiving social assistance** ^6^			
Not receiving	963 (86.1)		4,073,315 (98.4)^65^
Receiving	156 (13.9)		67,821 (1.6)
**Receiving disability benefits**			N/A
Not receiving	860 (72.8)		
Receiving	322 (27.2)		
**Difficulty living on income**			N/A
Very difficult	326 (19.4)		
Somewhat difficult	512 (30.5)		
Not very difficult	446 (26.6)		
Not at all difficult	394 (23.5)		
**Experience any discrimination in everyday life**			N/A
No	617 (36.9)		
Yes	1055 (63.1)		
**Overall health**			N/A
Poor	336 (20.3)	1183 (8.9)	
Fair	400 (24.2)	2566 (20.0)	
Good	503 (30.4)	4115 (30.6)	
Very good	322 (19.5)	3620 (25.9)	
Excellent	94 (5.7)	2027 (14.6)	
**ED visits in the past 6 months**	Range: 1 to 180, Mean: 3.22, SD: 10.286		N/A
One visit	793 (48.7)	(7701) 54.8	
More than one visit	834 (51.3)	(6029) 42.9	
**Have usual primary care home**			N/A
No	152 (9.1)	702 (95.0)	
Yes	1517 (90.9)	13,202 (94.0)	

### Latent Classes of Social and Economic Circumstances

LCA was conducted using Stata. Nine dichotomous social location variables were included in the model, and solutions in two to six classes were examined. The AIC and BIC suggested four classes, given that the AIC and BIC decreased from three to four classes (AIC: 21,184 to 20,486, BIC: 21,347 to 20,903), with very modest decrease for the five- (AIC: 20,585, BIC: 20,824) and six-class models (AIC: 20,486, BIC: 20,822). The final decision on the number of classes was based on interpretability; ultimately, we selected the six-class solution because it had greater explanatory power.

The social location indicators for each of the six classes are summarized in Table 3, with detailed demographics in Table 4. Being born in Canada or not and age were key features in each class. Class 1 was comprised primarily of people who were **younger, economically stable newcomers**^3^; all were employed and they tended to be younger (M = 42.6 years), to be predominantly male (57%), to be economically stable with stable housing, to not access shelters, and to have low financial strain. Class 2 were people who were **retired, economically stable, and born in Canada**; all were over 65 (M = 74.6 years), and most were retired (83.6%), had stable living conditions, had low financial strain, and did not access shelters. Class 3 was comprised of people who were **severely structurally disadvantaged, younger, and born in Canada**; this was the most economically compromised group, predominantly men (65%), younger (M = 43.3 years), all born in Canada, with the highest proportion of Indigenous people and the greatest shelter use (M = 41.6 nights among those who used a shelter in the past six months). Class 4 were people who were **unemployed, older newcomers**; they were older (M = 65.4 years), none were born in Canada, none were employed (68.5% retired), and they were predominantly women (57%) and less likely to have English as their first language, with stable housing, but variable financial strain. Class 5, the smallest class, was comprised of people who were **less employed, younger newcomers**; none were born in Canada, and only 24% were employed. They tended to be younger (M = 42.6 years), have stable housing, and have variable financial strain. This cluster included 16 people who identified with Indigenous groups from countries other than Canada (Australia, Guatemala, India, Mayan, Mestizo, Jamaica, Nigeria, and United States). Finally, Class 6 was comprised of people **under 65, born in Canada, with stable housing**. This was the largest class. All were under 65, all were born in Canada, 95% had English as their first language, they were predominantly women (56%), they had stable living situations, and none had used a shelter in the past six months, but had variable financial strain.

**Table 3.** Percent of People with Each Sociodemographic Indicator for the Six Latent Classes Based on Observed Values.

	Class 1 Younger, economically stable newcomers N = 320 (13.2%)	Class 2 retired, economically stable, born in Canada N = 405 (16.7%)	Class 3 severely disadvantaged, younger N = 313 (12.9%)	Class 4 Unemployed, older newcomers N = 233 (9.6%)	Class 5 less employed, younger newcomers N = 85 (3.5%)	Class 6 Younger, born in Canada, stable housing N = 1062 (43.9%)
Low financial strain (well off)	72.0	60.0	11.0	57.0	19.0	50.0
Stable living situation	100.0	93.0	39.0	97.0	55.0	98.0
Age > 65	3.0	100.0	5.0	67.0	0.0	0.0
Male gender	57.0	51.0	65.0	43.0	72.0	44.0
Born Canada	2.0	100.0	100.0	0.0	0.0	100.0
English first language	25.0	89.0	78.0	38.0	29.0	95.0
Indigenous	1.0	10.0	59.0	0.0	19.0	17.0
Employed	100	10.0	10.0	0.0	24.0	65.0
Shelter past year	0	0	56.0	0.0	29.0	0.0

Class 1: Younger, economically stable newcomers.

Class 2: Retired, economically stable, older people, born in Canada.

Class 3: Severely socially disadvantaged, younger, born in Canada.

Class 4: Unemployed older newcomers, English as second language, with variable economic situations.

Class 5: Not very employed, younger, male newcomers.

Class 6: Economically stable women under 65 years, born in Canada.

**Table 4.** Demographic Characteristics of the Six-Class Model.

Variable	Class 1 younger, economically stable newcomers	Class 2 retired, economically stable, born in Canada	Class 3 severely disadvantaged, younger males	Class 4 unemployed, older newcomers	Class 5 less employed, younger newcomers	Class 6 under 65, born in Canada, stable housing
	**n (%) of EQUIP ED Sample**					
**How difficult to live on income**						
Very or somewhat difficult	89 (28.2)	161 (39.9)	278 (89.4)	99 (42.9)	69 (81.2)	524 (49.7)
Not very/not at all difficult	1125 (71.6)	243 (60.1)	33 (10.6)	132 (57.1)	16 (18.9)	530 (50.3)
**Calculated age**	M = 42.6 (18-94) SD = 13.65	M = 74.6 (65-98) SD = 7.61	M = 43.3 (20-98) SD = 14.15	M = 65.4 (17-96) SD = 20.0	M = 42.6 (18-64) SD = 13.5	M = 42.1 (18-64) SD = 13.4
**Primary Work Status**						
Employed full or part-time	315 (100)	41 (10.2)	33 (10.6)	1 (0.4)	23 (27.1)	686 (65.5)
Seasonal, service, student, other	0 (0)	4 (1.0)	36 (11.6)	22 (9.5)	12 (14.1)	60 (5.7)
Unemployed	0 (0)	21 (5.2)	220 (70.7)	44 (51.8)	44 (51.8)	221 (21.1)
Retired	0 (0)	336 (83.6)	22 (7.1)	159 (68.5)	6 (7.1)	79 (7.5)
**Education**						
Less than high school	63 (19.7)	222 (54.8)	226 (72.2)	101 (43.5)	44 (51.8)	404 (38.1)
Completed secondary school/high school	49 (15.4)	67 (16.5)	39 (12.5)	41 (17.7)	9 (10.6)	268 (25.3)
Some college or more	206 (64.6)	114 (28.1)	47 (15.0)	87 (37.5)	32 (37.6)	386 (36.4)
Level not known	1 (0.3)	2 (0.5)	1 (0.3)	3 (1.3)	0	1 (0.1)

*Health Care Access: Having a Primary Health Care Home, Reason for Attending ED, and Acuity Rating*

As shown in Table 5, health care access varied with the classes. People in Class 3 (severely socially disadvantaged younger males) and Class 5 (unemployed newcomers) were significantly less likely to report having a primary care home and were significantly more likely to attend for an “ongoing health problem” as opposed to a new problem, an accident, or an injury than those in the economically more stable classes. The classes also varied on acuity at triage, with those who were more likely to have a primary care home (and thus more able to access primary care for lower acuity issues) presenting to ED with higher acuity problems. Class 2 (retired, economically stable, older people born in Canada) and Class 4 (unemployed, older newcomers) presented with somewhat higher acuity, perhaps reflecting access to primary care and the relationship between older age and serious health issues.

**Table 5.** Differences in Clusters by Having a Primary Care Home, Reason for Attending ED and Acuity Rating (CTAS).

	Class 1 younger economically stable newcomers 320 (13.2%)	Class 2 retired economically stable born in Canada 405 (16.7%)	Class 3 severely disadvantaged younger males 313 (12.9%)	Class 4 unemployed older newcomers 233 (9.6%)	Class 5 less employed younger newcomers 85 (3.5%)	Class 6 under 65 years, born in Canada, stable housing 1062 (43.9%)	p-value
Has Primary Care Home	87.7%	96.3%	80.4%	91.2%	81.7%	90.8%	<.001
Reason for Visit							
Accident or injury	25.8%	17.3%	18.3%	11.9%	23.6%	20.2%	<.001
New health problem	47.9%	40.1%	32.0%	45.2%	25.5%	38.6%	
Ongoing condition	26.3%	42.7%	49.7%	42.9%	50.9%	41.2%	
CTAS	3.17 (0.76)	2.89 (0.77)	3.20 (0.66)	2.89 (0.76)	3.14 (0.87)	3.13 (0.74)	<.001

Pairwise differences CTAS – 1 is higher acuity.

Class 1 is significantly different from class 2, 4, 6.

Class 2 is significantly different from class 1, 3, 6.

Class 3 is significantly different from class 2, 4.

Class 4 is significantly different from class 1, 3, 6.

Class 5 is not significantly different from any class.

Class 6 is significantly different from class 2, 4.

### Patients’ Self-Reported Experiences of Discrimination

For the overall sample, 63% reported experiencing some form of discrimination in their everyday lives; however, on a scale of 0 to 45, the mean was relatively low at 9.29 (SD = 10.3). The top reasons people thought they were discriminated against in their everyday lives, with more than 170 people (10% of the sample) identifying each, were appearance, age, race, ancestry, and gender. However, as shown in Tables 4 and 6, these experiences varied significantly with the classes, with the highest scores on the Everyday Discrimination Scale (M = 20.70, 0-45, SD = 13.52) being from those in Class 3 (severely socially disadvantaged and younger, including a large proportion of Indigenous people) and Class 5 (less employed, younger newcomers) (M = 12.88, 0-45, SD = 12.22) and lowest (M = 4.43, 0-36, SD = 6.74) for Class 2 (retired, economically stable, born in Canada).

**Table 6.** Differences in Clusters by Self-Reported Everyday Experiences of Discrimination, Experiences of Discrimination in EDs, Patient Ratings of Care, and Self-Reported Number of ED Visits Past 6 Months.

	Class 1 younger economically stable newcomers 320 (13.2%)	Class 2 retired economically stable born in Canada 405 (16.7%)	Class 3 severely disadvantaged younger males 313 (12.9%)	Class 4 unemployed older newcomers women 233 (9.6%)	Class 5 less employed younger newcomers 85 (3.5%)	Class 6 under 65, born in Canada, stable housing 1062 (43.9%)	p-value
Everyday discrimination Mean (SD)	6.27 (7.22)	4.43 (6.74)	20.70 (13.52)	5.06 (7.72)	12.88 (12.22)	10.21 (9.27)	<.001
ED discrimination (any) N (%)	44 (13.9%)	46 (11.0%)	143 (45.6%)	45 (19.5%)	41 (37.0%)	221 (20.8%)	<.001
Ratings of care Mean (SD)	8.39 (1.61)	8.88 (1.55)	7.59 (2.44)	8.51 (1.83)	8.48 (1.58)	8.33 (1.82)	<.001
Number ED visits past 6 months Mean (SD)	1.61 (1.69)	2.25 (2.47)	6.75 (13.18)	2.93 (12.46)	3.70 (9.33)	3.12 (10.37)	<.001

Pairwise differences Everyday Discrimination.

Class 1 is significantly different from class 3, 5, 6.

Class 2 is significantly different from class 3, 5, 6.

Class 3 is significantly different from every class.

Class 4 is significantly different from class 3, 5, 6.

Class 5 is significantly different from class 1, 2, 3, 4.

Class 6 is significantly different from class 1, 2, 3, 4.

Pairwise differences Number of ED visits.

Class 1 is significantly different from class 3.

Class 2 is significantly different from class 3.

Class 3 is significantly different from every class.

Class 4 is significantly different from class 3.

Class 5 is not significantly different from any class.

Class 6 is significantly different from class 3.

Pairwise differences Patient Ratings of Care.

Class 1 is significantly different than class 3.

Class 2 is significantly different from class 3, 6.

Class 3 is significantly different from every class.

Class 4 is significantly different from class 3.

Class 5 is significantly different from class 3.

Class 6 is significantly different from class 2, 3.

Similarly, for the overall sample, patient-reported experiences of discrimination during ED visits were low, with 21.5% reporting experiencing some form of discrimination during their ED visit. On a scale of 1 to 35, the overall average was 8.93 (Range: 7-35, SD = 4.132). The top five reasons participants thought they were discriminated against in the ED (each of which was identified by more than 50 people) were substance use, appearance, mental health, suspected of drug seeking, and age. Again, as shown in Table 6, a higher proportion of people in Classes 3 and 5 reported experiencing discrimination in the ED: 45.6% and 37%, respectively. This difference remained significant after controlling for everyday discrimination (*p* = 0.001). Patient-reported everyday experiences of discrimination were correlated at 0.372 with their reported experiences of discrimination during their ED visit.

### Patient Ratings of Care

Overall, patient ratings of care were high, with an average of 8.37 (SD = 1.862) on a scale of 1 to 10. The classes varied significantly on patient ratings of care, with the severely disadvantaged younger people (Class 3) providing the lowest ratings of care and differing significantly from all other classes. The significant differences between the classes on patients’ ratings of care remained after controlling for everyday discrimination (*p* = 0.032).

### Patients’ Self-Reported Number of ED Visits Within the Past Six Months

51.3% of participants reported having made more than one visit to the ED in the past six months, with an average of 3.2 visits in the past six months (cumulative range of visits: 1-180^4^, SD = 10.286). The classes varied on their number of ED visits in the past six months, with the severely structurally disadvantaged, younger people, Class 3, having the highest number of visits (m = 6.75, SD = 13.18) and being significantly different from every other class.

## Discussion

People's social and economic circumstances drive the need to seek help at EDs and their experiences of emergency health care. Our analysis clearly delineates six statistical classes of people based on the intersections of specific, pre-identified characteristics, and these groupings were strongly and consistently related to experiences of everyday and ED-specific discrimination, ratings of care, and use of the ED. The findings demonstrated how employment, housing stability, shelter use, and financial stability cluster together. It further demonstrates how age and being born in Canada or not are aspects of social location that may be particularly influential for PEOC and equity. Only one of the six classes was comprised of people both born in and born outside of Canada; similarly, three of the classes were comprised of people either under or over 65, two had less than 5% over 65, and only one (Class 4) had a mix.

Importantly, this analysis directly challenges the utility of any single category (such as gender, age, Indigenous identity, or being a newcomer) for examining equity, inequities, or experiences of care. For example, men predominated in Class 3 (severely disadvantaged, younger people, born in Canada) and Class 5 (less employed, younger newcomers); women predominated in Class 4 (unemployed, older newcomers) and Class 6 (born in Canada, under 65, with stable housing). This again suggests that being born in Canada or not is an important influence that intersects with gender. To take another example, although people with Indigenous identity were predominantly in those classes with the greatest financial strain and lowest housing stability (Classes 3 and 5), Indigenous people were classed with all clusters, including 17% in Class 6 (people under 65 years, with stable housing, born in Canada) and 10% in Class 2 (retired, economically stable, born in Canada), countering the tendency in public discourse to characterize Indigenous people as uniformly disadvantaged.

Overall, patients rated their ED care very highly, indicating little room to demonstrate change when using conventional measures of ratings of care. Research suggests that patients generally rate the care they receive in the ED as satisfactory^7,43^. However, the LCA allowed us to identify how various social and economic circumstances that people experience intersect and shape “who” is more likely to both seek help at EDs (because of their social circumstances) and rate their health care experiences in EDs more poorly. Thus, these findings suggest directions for more tailored improvements in care delivery, guiding organizations to consider how health care is influenced by social and economic circumstances, and thus perpetuating inequities, and for whom such improvements are urgently needed. Our analysis also extends understanding of the ways that persistent and deepening inequities in Canada create conditions in which people experiencing significant hardships need to seek help at EDs. These insights prompt reconsideration of the role and responsibility of EDs in serving people who are most in need, given the socioeconomic contexts of people's lives.

This analysis also provides important insights into patients’ perceptions of their experiences of discrimination during ED visits. The low correlation suggests that not all people who experience discrimination in their everyday lives experience discrimination during ED visits. The classes comprised of older people (Classes 2 and 4: older people born in Canada and newcomers, respectively) reported the lowest everyday discrimination, yet reported experiencing ED discrimination. Aligning with literature showing that people who experience structural disadvantages experience stigmatization from ED staff when seeking care^6,7,44–46^, this analysis illustrates further that structural inequities intersect and are associated with significantly higher ratings of discrimination, in both the everyday and the ED context.

The LCA illustrates how an intersectional lens necessitates considering who might be overlooked in the understanding of how structural inequities operate in people's lives, as well as how that shapes their need to seek care at EDs and their overall access to care. Accessible primary care has long been identified as critical to health care delivery and, more specifically, to support reducing the need for ED services and urgent care^9,10,15,47^. The LCA demonstrates that the issue of accessible primary care that aligns with people's interrelated health and social needs is highly complex and requires an intersectional understanding to fully comprehend how social circumstances and primary care access are intertwined.

Importantly, this analysis sheds light on multiple, intersecting factors that may be influencing repeated visits to EDs. To date, analyses of repeat ED use have focused on the characteristics of the people who account for that use, not necessarily the structural conditions of their lives nor how health care is structured to respond to people's needs. Recognizing the heterogeneity of people making repeat ED visits, efforts have been made to identify subgroups of people. For example, a recent Canadian analysis identified high users of EDs as “the elderly,” “mental health and alcohol use,” “young mental health,” and “short term” (people who made regularly spaced visits over a short period of time for problems such as urinary tract infection, follow-up examination, pyelonephritis, and abscess)^48^. Another study examined people over age 65 and grouped people as “low comorbidity,” “people with cancer,” “people with pulmonary and cardiac diseases,” and “people with dementia or mental health disorders”^49^. In our analysis, repeat visits were largely accounted for by people in Classes 3 and 5, the people who were less likely to have a primary care home and were facing the greatest economic and housing instability. This supports the importance of policy and funding efforts toward: (*a*) enhancing primary care capacity and responsiveness, especially models of interprofessional, team-based care tailored to serving those with barriers to accessing care^50^ and those who experience stigma and intersecting forms of discrimination^15,51^ and, importantly, (*b*) linking patients to social agencies for housing and income support. Developing the capacity, resources, and time to directly and immediately link patients to housing support and related services could be framed as aligning with efficiency goals related to responsiveness^15^. These classes were also comprised of the people experiencing the greatest housing instability and financial strain, suggesting that efforts to relieve strain on EDs must include broader structural efforts for addressing housing and homelessness. Finally, those with the greatest number of repeat visits were also those reporting the highest levels of discrimination in EDs, suggesting that efforts to reduce stigma and discrimination in EDs must be prioritized.

Reducing the impact of stigma and discrimination within health care settings, including EDs, is within the purview of health care organizations. Tackling stigma and discrimination has potential to improve patient experiences and outcomes and to improve system efficiency and effectiveness. A recent meta-synthesis of patient experiences in EDs showed that subjective positive experiences are associated with better clinical effectiveness and patient safety, including lower mortality and morbidity^52^. Our research on how to promote equity in health systems^20,21,32,50,51,53–55^, together with research on stigma, show that to achieve these improvements and reduce stigma^56–60^ requires: (*a*) making such efforts a priority at every level of the organization, (*b*) including and going beyond training and education for staff (eg, on implicit bias, use of non-stigmatizing language) to catalyze structural changes in how care is organized and enacted, and (*c*) measuring patient experiences, staff experiences, shifts in organizational processes and cultures, and stigma-sensitive indicators routinely and over time. To this end, we have created Action Kits aimed at supporting health care organizations to implement organizational change toward equity and destigmatization (https://equiphealthcare.ca/). These kits include tools to facilitate interprofessional staff discussions (eg, “Rate Your Organization), illuminate and evaluate taken-for-granted stigmatizing processes (eg, An Equity Walkthrough), and plan and evaluate point-of-care and organizational-level changes. In the ED context, for example, these have been used to see how triage physical layout and processes, signage, waiting areas, intake processes, and security and surveillance can be improved to create environments that are more intentionally welcoming, particularly for people who often experience stigma^61^.

Using an equity lens and equity-oriented data collection methods fostered greater inclusivity in our sample compared to data routinely collected on PEOC. However, we knew at the outset that some patients presenting could not consent to participate due to an altered level of consciousness or to their acuity; thus, we were not able to capture the entire population of patients presenting for care. The people we lost to follow up potentially are those who experience greater structural disadvantages: significantly more people who were not born in Canada, people with English as a second language, those with precarious housing, and those who had accessed a shelter in the past six months. However, this serves to make our analysis more conservative.

Our analysis is also limited by the limitations inherent in the measures we used, particularly our measures of discrimination. As Scheim and Bauer^37^ note, most measures of discrimination, including the one we used, were developed initially to study ethno-racial discrimination. Further, the Everyday Discrimination Scale^38^ invites respondents to attribute the motives of others, which a few of our participants found difficult. A number of participants wanted to attribute the discrimination they experienced to the person enacting the discrimination, not to their personal characteristics. Finally, because everyone was invited to answer the questions and identify all potential reasons, we had a number of people who identified as “white,” “European,” or “Caucasian” identify “skin color” as a reason for experiencing discrimination in their everyday lives, reflecting the narrative of “reverse racism” that is part of the wider social discourse in Canada. These complexities highlight the need for ongoing research and conceptual clarification to further develop robust intersectional discrimination measures^37^.

In summary, this analysis showed how an equity lens and intersectional analysis help to illuminate the relationships between structural disadvantage and care experiences. One of the key challenges was to analyze the data in ways that would show who was experiencing the greatest barriers to care, including perceived discrimination and stigma, without essentializing or potentially stigmatizing or pathologizing particular groups as “problems” or as “overusing” the ED, reducing individuals to single categories or promoting potentially adversarial relations between staff and patients (eg, by reporting ratings of care by groups defined by single categories). The LCA provided an approach to doing so consistent with an intersectional approach.

*Ethics—*The study protocol has been approved by the research ethics boards of the University of British Columbia and the University of Northern British Columbia (UNBC) and the research ethics boards of Fraser Health Authority, Northern Health Authority, and Providence Health Care (approval numbers H16-03397, H17-01548, and H18-01423).

Colleen Varcoe, PhD, is a professor in the School of Nursing at the University of British Columbia (UBC). Past positions include Director, School of Nursing, UBC (2011-2012), and associate professor, School of Nursing, UBC (2005-2010). Her educational background is: PhD, UBC (1997); M.Sc., UBC (1994); M.Ed., UBC (1987); and B.S.N., UBC (1979). Dr Varcoe is a leader in research on violence and inequity, with emphasis on women's and Indigenous people's health. Her research is currently focused on interventions to mitigate the health effects of violence for women who have experienced partner violence and on interventions to help health care settings, such as EDs, promote equity. She is a leader in implementing and studying equity-promoting health care, including trauma- and violence-informed approaches, cultural safety, and harm reduction.

Annette Browne, PhD, is a professor in the School of Nursing at the University of British Columbia (UBC). Past positions include associate professor, School of Nursing, UBC (2005-2011), and assistant professor, School of Nursing, UBC (2002-2005). Her educational background is: PhD, UBC (2003); M.Sc., University of Rhode Island (1992); and B.S.N., University of Manitoba (1986). Dr Browne studies health and health care inequities, with a particular focus on health inequities affecting Indigenous peoples. She conducts research on strategies to enhance equity-oriented health care for Indigenous and non-Indigenous people, including interventions to address systemic racism and discrimination and to support the uptake of cultural safety and trauma- and violence-informed care. Her work is aimed at promoting health equity through improvements in nursing practice, health care delivery, and health policy.

Vicky Bungay, PhD, is a professor in the School of Nursing at the University of British Columbia (UBC). Past positions include associate professor, School of Nursing, UBC (2014-2019), and assistant professor, School of Nursing, UBC (2009-2014). Her educational background is: PhD, UBC (2008); M.Sc., Dalhousie University (1994); and B.S.N., St. Francis Xavier University (1989). Dr Bungay's research focuses on addressing inequities that negatively affect people's health and well-being, including the devastating effects of stigma, discrimination, and violence. She explores how research partnerships can positively impact communities that are excluded in health and social policy; programming that affects their lives; and how community-based interventions support real-world evidence.

Nancy Perrin, PhD, is the Director of Biostatistics and Methods Core at Johns Hopkins School of Nursing. Past positions include Director of Biostatistics Core, Center for Health Research (2011-2018), and Senior Director of Research, Data, and Analysis, Center for Health Research (2013-2015). Her educational background is: PhD, Ohio State University (1986); M.A., Ohio State University (1983); and B.A., University of California (1980). Dr Perrin conducts research in the area of violence against women both in the United States and in conflict countries of Somalia, South Sudan, and Democratic Republic of the Congo. She is developing a measure of social norms around violence against women, has conducted a national prevalence survey of violence against men and women in Somalia, and has conducted an effectiveness trial for a social norms intervention in Somalia and South Sudan.

Erin Wilson, PhD, N.P.(F.), is an assistant professor in the School of Nursing at the UNBC and a family nurse practitioner at Northern Health. Past positions include assistant professor (TERM), School of Nursing, UNBC (2010-2018), and community health nurse with the Government of Yukon (2006-2007). Her educational background is: PhD, UNBC (2017); M.Sc., UBC (2006); and B.S.N., University of Manitoba (2000). Her research examines delivery of primary health care in rural settings. She draws on patient-oriented and interpretive approaches to examine how interprofessional teams can improve access, coordination, and continuity of care in ways that are safe and equitable for patients and communities

Nadine Wathen, PhD, is full professor and Canada Research Chair in Mobilizing Knowledge on Gender-Based Violence in the Arthur Labatt Family School of Nursing at Western University, and Academic Director of the Centre for Research on Health Equity and Social Inclusion. Past positions include Graduate Chair, Health Information Science Program, Western University (2020-2021 and 2011-2015), and assistant professor, Faculty of Information Studies, University of Toronto (2005-2007). Her educational background is: post-doctoral fellowship, McMaster University (2004-2006); PhD, Western University (2004); M.A., Western University (1992); and B.A., Honors (First Class), Dalhousie University (1990). Her research examines the health and social service sector response to gender-based violence, interventions to reduce health inequities, and the science of knowledge mobilization. A particular focus is developing person-centered interventions that enhance health equity and take a gendered, trauma-, and violence-informed approach to providing services for those experiencing violence and/or marginalization. She is deeply committed to a partnership approach to research and knowledge sharing and has led a number of federally funded research initiatives, as well as international research and knowledge mobilization networks. She has more than 100 publications in health care, health and public policy, and information science journals and three edited collections. See www.nadinewathen.ca.

Dr David W. Byres is the Interim President and CEO of the Provincial Health Services Authority. He has more than 25 years of experience in the health sector, most recently as the associate Deputy Minister, Clinical Leadership for the B.C. Ministry of Health. Previously, he served as the Executive Vice President for Providence Health Care and later as the Chief Nurse Executive for the Ministry of Health. His educational background is: PhD in nursing practice from American Sentinel University (2016); M.S.N. from University of British Columbia (UBC) (2006); B.S.N. from University of British Columbia (1997); and B.A. in psychology from the University of Victoria (1987). He is an adjunct professor with the School of Nursing at UBC and adjunct assistant professor with the School of Nursing at the University of Victoria. He is a Certified Health Executive with the Canadian College of Health Leaders and recipient of the Dean's Medal of Distinction from the UBC Faculty of Applied Science and, he was recently inducted as a Fellow of the Canadian Academy of Nursing.

Elder Roberta Price (Coast Salish - Snuneymuxw and Cowichan Nations) is the elder for Critical Research in Health and Health Care Inequities, School of Nursing, University of British Columbia (UBC). She is passionate about creating shared spaces for both Indigenous and Western approaches to healing and health. Past positions include: Elder Advisor to Governing Council, BC Network Environments for Indigenous Health Research (2020); board co-chair at Kilala Lelum (2018); Elder Advisor for the Department of Advanced Education at Simon Fraser University (2016); and principal investigator for the Department of Psychiatry in the Faculty of Medicine at UBC (2015). In March of 2021, Elder Roberta Price received an Honorary Doctorate of Law degree from UBC.

## Notes

1. Structural factors or influences can be conceptualized as the totality of ways in which societies sustain social and health inequities through policies and practices enacted through reinforcing systems of housing, education, employment, earnings, benefits, credit, media, health care, and criminal justice. These social dynamics, patterns, and practices reinforce and recreate inequitable distribution of resources, including access to health care and social supports (^62^). To say that social and economic inequities, gendered inequities, racism, and other forms of discrimination are structural is to imply that they exist in the institutions and social practices of our society and cannot be explained as merely situational (^30^).

2. In Canada, Indigenous people refers to the diversity of populations in Canada, which includes First Nations, Métis, and Inuit people (^63^).

5. The response options included in “precarious housing” are: couch-surfing, shelter, on the street, in vehicle (car or van), SRO, rooming house, RV or trailer, Tent, and other.

6. In BC, a single person on income assistance receives $935 each month, while a single person on disability assistance receives $1,358.42.

3. “Newcomers” is used as a preferred term to indicate people who were not born in Canada; this includes people classified by the Canadian federal government as immigrants or refugees.

4. Four people were required to visit EDs daily to receive medications.
